# The Evaluation of the Efficacy and Safety of Oral Colchicine in the Treatment of Knee Osteoarthritis: A Meta-Analysis of Randomized Controlled Trails

**DOI:** 10.1155/2022/2381828

**Published:** 2022-01-29

**Authors:** Weijie Liu, HaoChen Wang, Chao Su, Shida Kuang, Yilin Xiong, Yusheng Li, Shuguang Gao

**Affiliations:** ^1^Department of Orthopaedics, Xiangya Hospital, Central South University, Changsha, Hunan Province, China; ^2^Hunan Key Laboratory of Joint Degeneration and Injury, Changsha, Hunan, China; ^3^Hunan Engineering Research Center of Osteoarthritis, Changsha, Hunan, China; ^4^National Clinical Research Center of Geriatric Disorders, Xiangya Hospital, Central South University, Changsha, Hunan, China

## Abstract

**Objective:**

To evaluate the efficacy and safety of oral colchicine in the treatment for knee OA.

**Design:**

Meta-analysis. *Data Sources*. Embase, PubMed (MEDLINE), the Cochrane Library, and Web of Science from inception to December 12, 2021. *Study Selection*. RCTs comparing colchicine with placebo for knee OA were included. No language or date restrictions were applied. Two authors abstracted data and determined quality. Outcomes of interest included VAS-pain, WOMAC total index, and patient-reported adverse events.

**Results:**

A total of five RCTs including 400 adult patients with OA met the inclusion criteria. The mean age of patients included was 56.05 years (range 21 to 79), and 80.87% were female. There was no difference in VAS-pain (MD -1.49; 95% CI -3.15, 0.17; *p* = 0.08) when compared colchicine group with placebo group. And there was no statistically difference in WOMAC total index (std. MD -0.13; 95% CI -0.64, 0.38; *p* = 0.61) and patient report adverse events (RR 1.23; 95% CI 0.72, 2.11; *p* = 0.46).

**Conclusion:**

Colchicine is not currently recommended as a treatment for knee OA but might have insignificant effect. The conclusion is limited due to the variation in assessment indicator among available data. Further RCTs with larger sample size and longer follow-up are needed to confirm the findings.

## 1. Introduction

Osteoarthritis (OA) is a multicausal, chronic disabling disease involving all joints, accompanied by lesions of articular cartilage, subchondral bone, ligaments, synovium, joint capsule, and muscular structures around joints [1]. About 10-17% of adults were found to be present with knee OA over 40 years old, and a half of adults over 60 years old [2–7]. In addition, women have a higher prevalence and greater disability rates than men in this disease [2, 3]. OA is a leading cause of disability, and it is expected to be a main cause of years lived with disability globally [8]. The social-economy impact of OA cannot be ignored as OA not only leads to the decline of patients' physical function, quality of life, and social participation but also brings a huge burden to society [9]. Knee joint is the most common site of OA, and the main causes of knee OA are aging, female sex, previous injury, and obesity [4, 5, 9]. With the aging of the population and the increasing proportion of obese people in current society, we have an unmet need to find effective ways to treat OA [9].

Currently, there are many drugs for treating OA, but most of them have inaccurate effects and limited effectiveness [10]. The effectiveness of colchicine in preventing inflammation caused by calcium crystals, such as treating gout and pseudogout, has been widely recognized [11]. It has been reported that uric acid can activate the innate immune response involved in OA [12]. Accordingly, it is hypothesized that colchicine may have the potential to treat OA, especially knee OA [13–16]. However, there was a controversy about whether colchicine is effective in treating osteoarthritis recently [12, 17–21]. Therefore, the purpose of this meta-analysis is to evaluate whether oral colchicine is effective and safe to treat knee OA, comparing with placebo according to the visual analog scale for index knee pain (VAS-pain), Western Ontario and McMaster Universities (WOMAC) total index, and patient-reported adverse events, using individual patient data from published trails.

## 2. Methods

### 2.1. Search Strategy

This study was based on the Cochrane Review Methods [22]. Adhering to the guidelines of the updated Preferred Reporting Items for Systematic Reviews and Meta-analyses (PRISMA) statement [23], systematic literature searches were undertaken using PubMed (MEDLINE), EMBASE, the Cochrane Library, and Web of Science, from inception to December 12, 2021, for studies that compared the outcome of VAS-pain, WOMAC total index, and patient-reported adverse events. There were no restrictions on language, year, or type of publication. The title, abstract, Mesh, and keywords fields search terms were used included “colchicine” [Mesh] AND “osteoarthritis”[Mesh]. Manual searches were also performed for articles potentially missed by the electronic search. A full list of terms selected for searching the electronic databases is presented as supplemental information (Supplementary Table S1).

### 2.2. Study Selection

Two investigators (W.L. and H.W.) identified the titles and abstracts of the retrieved papers and selected relevant studies for a full review independently. If suitability could not be determined, the full article was evaluated by a discussion with a third author. Studies were included in the meta-analysis if (1) they included patients who suffered knee pain and there has support evidence of diagnosing as knee OA, likewise, the presence of joint space narrowing, subchondral sclerosis, and osteophyte in their radiographic studies, or there has laboratory findings suggestive of OA; (2) they evaluated the severity of osteoarthritis with VAS-pain or WOMAC index at the end of the study and also reported the adverse events; (3) completed reported parameters, including means, standard deviations or standard error, and sample size in each group (colchicine and placebo group); (4) the article category is randomized controlled trial (RCT); (5) clinical trial have outcome; (6) used adequate statistical methods to compare the amount and proportion of clinical outcomes between two groups.

### 2.3. Data Extraction

Two authors (W.L. and H.W.) independently recorded the following data from included study using a predefined data extraction form: (1) means and standard deviations of patient-reported outcome: VAS-pain (standardized to 0-10 cm) and WOMAC index; higher scores reflected more symptoms, and poorer physical function; (2) the population of adverse events in each group (N_1_) and total adverse events in each group (N_2_): N_1_ multiply *n* (types of adverse events). If these variables mentioned above were not acquired in the articles, the study authors would contact by email to request the data from the article author. Any disagreement unresolved by discussion was reviewed by a third author (C.S.) if needed. If studies only reported outcome data in pictures, Engauge Digitizer software (12.1) was used for data extraction. If studies do not report endpoint but baseline and change outcome, endpoint data was calculated using the formula referenced by the Cochrane handbook (chapter 16.1.3.2; version 5.1.0).

### 2.4. Assessment of Methodological Quality

**Figure 1:**
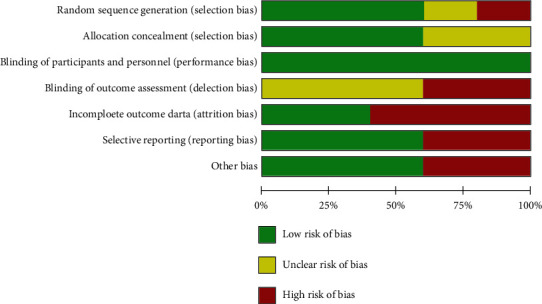
Risk-of-bias graph.

**Figure 2:**
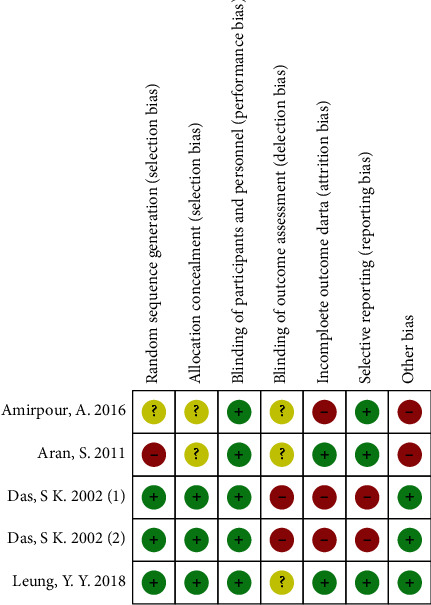
Risk-of-bias summary.

Two authors (C.S. and Y.L.) independently assessed the methodological quality of each study using the risk of bias table referenced in Cochrane handbook [22]: including rating of the random sequence generation; allocation concealment; blinding of participants and personnel, and blinding of outcome assessment; selective outcome reporting; incomplete outcome data, and other bias. Independent evaluation of included RCTs was marked as rank of high, low, or unclear risk of bias in each methodological quality items (Figures 1 and 2). Any unresolved differences of quality assessment between the two authors were resolved by consensus or by consultation with a third author (C.S.). Publication bias was not assessable in these trials because there are five studies included in the meta-analysis, which have not yet met the minimum publications requirement of 10 [22].

### 2.5. Data Synthesis and Statistical Analysis

The means and standard deviations of VAS-pain and WOMAC (total and modified total) were entered in RevMan to carry out the meta-analysis. The main outcomes of the meta-analysis were the endpoint of mean difference (MD) using random effect in VAS-pain and risk difference using random effect of adverse events. Due to the inconsistent total score of total WOMAC score and modified WOMAC score, standard mean difference (std. MD) was used in the data synthesis of WOMAC total. For all comparisons, risk difference (RR) and 95% confidence intervals (CI) were calculated for binary outcomes, while mean difference, std. MD, and 95% CI were calculated for continuous outcomes. *I*^2^ statistic was used to evaluate the heterogeneity of between-study. As reference by previous meta-analysis, when *I*^2^ with values under 25%, the heterogeneity of between-study considered low, when with value upper 25% and under 50%, the heterogeneity of between-study considered moderate, and when with value upper 25% and under 75%, the heterogeneity of between-study considered high. All statistical analyses were performed using RevMan version 5.3.

### 2.6. Patient and Public Involvement

Patients and/or the public were not involved in the design, conduct, reporting, or dissemination plans of this research.

## 3. Results

### 3.1. Study Identification, Characteristics, and Methodological Quality

**Figure 3:**
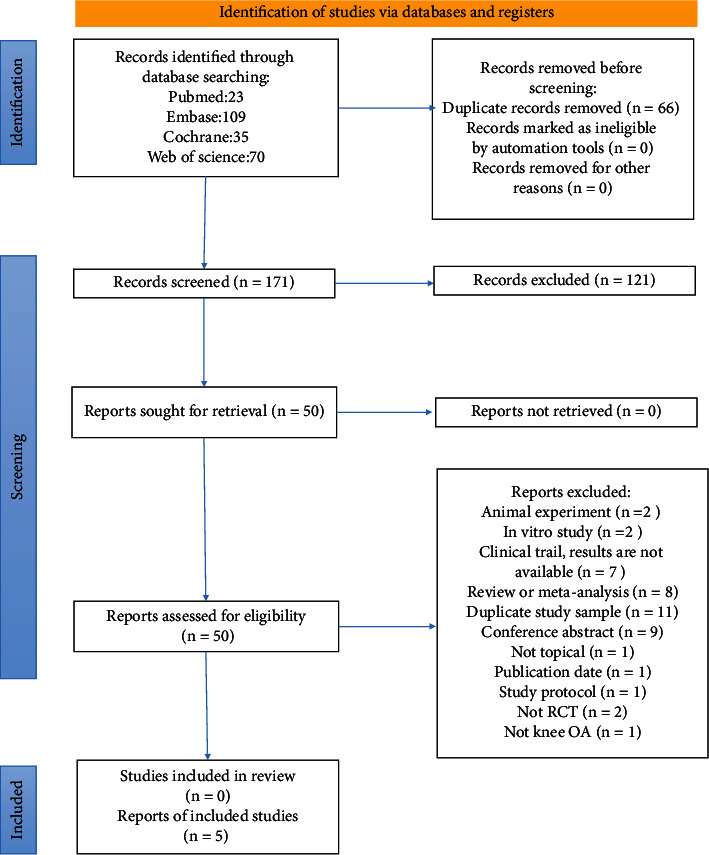
PRISMA (Preferred Reporting Items for Systematic Reviews and Meta-Analyses) 2020 flow chart, depicting study selection. Databases were searched for articles published from inception until December 12, 2021.

Figure 3 shows the details of study identification, inclusion, and exclusion. The database searches returned 143 citations after removal of duplicates. Five studies met the criteria for inclusion in the meta-analysis (Figure 3). The age range of the participants in included studies was 21 years to 79 years old, with the minimum 3 month and maximum 5 months follow-up (Table 1). Five RCTs prospectively compared VAS-pain (Table 1) and four prospectively compared WOMAC function, total WOMAC, or modified WOMAC index (Table 1) between colchicine and placebo group, and all five studies reported the amount and types of adverse events during the trails (Table 1). Three studies did baseline therapy, using drugs like naproxen [20]; piroxicam and methyl prednisolone acetate injection [12]; topical analgesics; supplements, and nonsteroidal anti-inflammatory drugs (NSAIDs) [12, 20, 21]. All five studies have a high female ratio with Amirpour et al.'s [20] study up to 98% and Aran et al.'s [19] up to 100%. Two studies [12, 18] included patients with CPPD (calcium-pyrophosphate-deposition-disease), two [19, 20] were not, and one [21] was not mentioned. Major complications were reported among all the trials, and one trail reported no serious adverse events happened. The conclusion of four studies supported that colchicine was effective in treating knee OA while Leung et al.'s [21] study came to the opposite view that colchicine was lack of efficacy compared with placebo. As two of the five included RCT studies [12, 19] had no baseline data, we gave up doing meta-analysis of variation value and performing meta-analysis on the endpoint of VAS-pain (standardized to 0-10 cm) and endpoint of WOMAC total index. Das^‡^ et al. [18] reported total WOMAC with total score of 96; Amirpour et al. and Das^†^ et al. [12] reported total modified WOMAC index with total score of 108; Leung et al. reported total WOMAC index with total score of 100, thus, we used std. MD in the data synthesis of WOMAC total. A risk-of-bias graph (Figure 1) and summary (Figure 2) were prepared to depict the Cochrane bias parameters against which the studies were judged.

**Figure 4:**
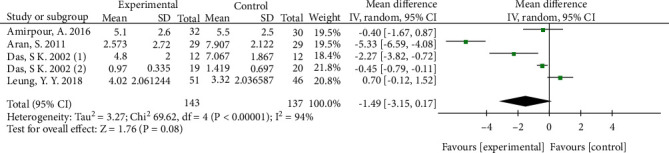
Endpoint of VAS-pain.

All five studies compared endpoint of VAS-pain in colchicine and placebo group. The pooled MD in endpoint of VAS-pain was -1.49 (95% CI -3.15, 0.17; *p* = 0.08; *I*^2^ = 94%; Figure 4), indicating that there is an insignificance difference in favor of colchicine in drug efficacy evaluated by VAS-pain between colchicine and placebo group.

**Figure 5:**
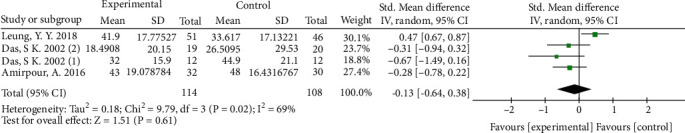
Endpoint of WOMAC total index.

Four studies compared endpoint of WOMAC total index in colchicine and placebo group. The pooled std. MD in endpoint of WOMAC total index was -0.13 (95% CI -0.64, 0.38; *p* = 0.61; *I*^2^ = 69; Figure 5), indicating that there is no statistics difference in drug efficacy evaluated by WOMAC total index between two groups.

**Figure 6:**
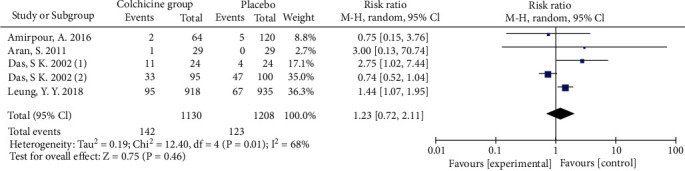
Endpoint of patient-reported adverse events.

All five studies' patients reported the adverse events and the pooled RR with value of 1.23 (95% CI 0.72, 2.11; *p* = 0.46; *I*^2^ = 68; Figure 6), indicating that there is also no difference in adverse events occurring between two groups.

## 4. Discussion

The main finding of this meta-analysis was that oral colchicine might reduce knee pain of OA patients insignificantly. And it would not be helpful for improving knee pain, stiffness, and function. This is contrary to the recommendation in favor of oral colchicine that can improve pain severity supported by four studies [12, 18–20] and physical function supported by other three [12, 18, 20]. Nevertheless, oral colchicine is relatively safe compared with placebo, which oral colchicine 0.5 mg twice daily may be a secure dosage for a patient to treat knee OA.

VAS-pain and WOMAC total index were selected as primary outcome measures, as the VAS is a reliable, valid, responsive, and frequently used pain outcome measure; and the WOMAC index is the most often used outcome in research about OA, especially in the lower limb and multiple studies have tested the WOMAC OA index for validity, reliability, feasibility, and responsiveness to measure changes after different interventions of OA patients [24–26]. Colchicine can reduce the symptom of patients' knee pain, stiffness, and function in four studies [12, 18–20] which reported a reduction of both indicators, but no statistical difference was found between two groups. With regard to safety, subjects in 5 studies [12, 18–21] took colchicine 0.5 mg twice daily. Considering VAS-pain, no difference in favor of colchicine was found (*p* = 0.08). After we excluded the Aran's outcome from data, the outcome of *I*^2^ was decreased from 94% to 76% (*p* = 0.34), indicated that the outcome of VAS-pain from Aran's might be the source of heterogeneity of the outcome. This finding could be explained by several factors. It could be due to variation between the included studies in methodology, primary outcome, and basic construct of the given treatment. Besides, only the patient-reported adverse events were directly comparable across the trials. When we performed the systematic literature search again in December, we found that Davis et al.'s [27] study reported 64 adult patients (54 females, 10 males) aged 40-80 years old with hand OA were randomized to receive colchicine (0.5 mg twice daily) or matching placebo for 12 weeks. They found that colchicine cannot reduce hand pain recorded on VAS, laboratory examination outcome like C-reactive protein, tender and swollen joint count, and Michigan Hand Questionnaire total, function, and pain scores etc., as no difference was found between colchicine and placebo groups. Thus, the results of Davis et al.'s [27] study do not support colchicine for treating symptomatic hand OA.

Basic calcium phosphate (BCP) crystal is detected in the synovial fluid of OA patients, and hydroxyapatite is the most common form found in OA joints [28]. Studies have shown that there is a positive correlation between synovial fluid BCP crystal levels and radiographic OA severity, and colchine has been successfully used in the treatment of BCP crystal deposition disease [29, 30]. In Denoble et al.'s study [31], quantitative radiology and scintigraphy showed that uric acid is a marker of OA severity and suggested that uric acid may be a factor that promotes the pathological process of OA by activating inflammasome. Synovial fluid uric acid, which could be seen in patients with gout, can aggravate OA symptoms by upregulating the expression of IL-1*β* and IL-18, which recent trails involved colchicine to treat OA as it seems to block two cytokines release by inhibiting NLRP3 (Nacht, leucine-rich repeat and pyrin domain containing protein 3) inflammasome [30–32]. Calcium pyrophosphate dihydrate (CPPD) crystals also have been found to activate the NLRP3 inflammasome but required about over 10 times amount of CPPD to activate. Chronic CPPD crystal arthritis is similar to OA in clinic, sometimes they are occurring in patients' joint combined, but they are not related [12]. However, the existence of CCPD may have an impact on the evaluation of OA patient's VAS-pain and WOMAC total index. Though pooled data showed that colchicine lacked efficacy in reducing patient's knee pain and may be useless in improving knee pain, stiffness, and function, colchicine has the molecular mechanism above. Thus, we should treat the outcome with caution, as colchicine may have insignificant effects on pain relief and function improvement in OA patients.

Limitations of this meta-analysis are present: first of all, no correction for potential confounders such as baseline treatment, age, gender, BMI, or length of follow-up could be performed due to the relatively limited number of patients, and BMI was available only in two studies [20, 21], which could act as important confounders influencing the outcomes, as the weight factor is an important part of the formation and development of knee osteoarthritis [33, 34]. Second, it should be mentioned that the research of Das^†^ [12] was a well-performed study, and more of these studies are needed. Sincere attempts were made to contact the authors of the three articles [12, 18, 19] to obtain WOMAC subscale index for data synthesis but received no reply. Third, regarding the data synthesis, two studies [19, 20] excluded subject with CPPD which may interfere with the results, two [12, 18] were not, and one [21] was not mentioned. Lastly, in all of these studies, the sample size was small and long-term studies are required to determine the duration of treatment, side effects, exact drug dose, efficacy, and safety, which means more of long-term and large sample size RCTs are needed to verify our findings. Despite these limitations, this study is the first to provide a meta-analysis of the efficacy and safety of oral colchicine in the treatment of knee OA.

## 5. Conclusions

Colchicine is not currently recommended as a treatment for knee OA, as the meta-analysis found that oral colchicine might reduce knee pain insignificantly and cannot significantly improve knee pain, stiffness, and function. Adverse events found no difference in the occurrence of between colchicine and placebo group. Larger and longer-term RCTs excluding CPPD and using validated outcome measures are needed to confirm the findings.

## Figures and Tables

**Table 1:** Basic characteristics of included studies.

Author; year	LEO	*N* at baseline	Female (%)	Mean age ± SD	Grade of OA	Dose	Primary outcome measures	Adverse events (*N*)	Follow-up (month)
Amirpour et al. 2016 [20]	I	81	98	58 ± 9.00	Not mentioned	0.5 mg/bid	VAS; modified WOMAC index	C: 2; P: 5	4
Aran et al. 2011 [19]	I	61	100	60.15 ± 7.47	Primary OA	0.5 mg/bid	Patients' global assessment; physician's global assessment (recorded on VAS)	C: 1; P: 0	3
Das et al. 2002^‡^ [18]	I	36	77.8	53.5 ± NA	Moderately severe symptomatic OA	0.5 mg/bid	Index knee pain (VAS); total WOMAC scores.	C: 11; P: 4	5
Das et al. 2002^†^ [12]	I	39	66.7	52.91 ± 8.08	Not mentioned	0.5 mg/bid	Vas pain; Total KGMC scale (modified WOMAC index suit for India)	C: 33; P: 47	5
Leung et al. 2018 [21]	I	109	70.7	58.48 ± 8.67	0-IV (KL grade)	0.5 mg/bid	Total WOMAC score; improvement in pain (VAs);	C: 95; P: 67	4

LOE: level of evidence; *N*: number; SD: standard deviation; NA: not applicable; OA: osteoarthritis; KL: Kellgren and Lawrence grading system; WOMAC: Western Ontario and McMaster University Osteoarthritis Index; VAS: visual analog scale; C: colchicine group; P: placebo group.

## Data Availability

The datasets used or analysed during the current study are available from the corresponding author on reasonable request.

## Additional Points

*Provenance and Peer Review*. This study is not commissioned and externally peer reviewed.

## Supplementary Materials

Supplementary Materials Supplementary Table S1: search strategy.
