# Supplementary material for: The Evaluation of the Efficacy and Safety of Oral Colchicine in the Treatment of Knee Osteoarthritis: A Meta-Analysis of Randomized Controlled Trails
Source: Biomed Res Int. 2022 Jan 29;2022:2381828. doi: 10.1155/2022/2381828 (PMC8817842; doi:10.1155/2022/2381828)
Supplement: Supplementary Materials — Supplementary Table S1: search strategy. [file 2381828.f1.docx]

**The evaluation of the efficacy and safety of oral colchicine in the treatment of knee osteoarthritis:**

**a meta-analysis of randomized controlled trails**

**Authors:** Weijie Liu^1*^, Haochen Wang^1*^, Chao Su^1^, Shida Kuang^1^, Yiling Xiong^1^, Yusheng Li^1^, Shuguang Gao^1,2,3,4^

*Weijie Liu and Haochen Wang contributed equally

**Authors affiliations:**

1. Department of Orthopaedics, Xiangya Hospital, Central South University, Changsha, Hunan, China;
2. Hunan Key Laboratory of Joint Degeneration and Injury, Changsha, Hunan, China;
3. Hunan Engineering Research Center of Osteoarthritis, Changsha, Hunan, China;
4. National Clinical Research Center of Geriatric Disorders, Xiangya Hospital, Central South University, Changsha, Hunan, China.

**Address for correspondence:** Shuguang Gao, MD, Department of Orthopaedics, Xiangya Hospital, Central South University, No. 87 Xiangya Road, Changsha, Hunan, China, 410008 Tel: +8613875980341; Fax: 0731-84327332, E-mail: [251469675@qq.com](mailto:251469675@qq.com)

**Supplementary Table S1.** **Search Strategy**

| PubMed | #1: ((((((((((((((((((((((("Colchicine"[MeSH Terms]) OR (colcemid*[Title/Abstract])) OR (demecolcine[Title/Abstract])) OR (colchamine[Title/Abstract])) OR (lumicolchicine*[Title/Abstract])) OR (gamma‐lumicolchicine*[Title/Abstract])) OR (beta‐lumicolchicine[Title/Abstract])) OR (colchicin*[Title/Abstract])) OR (colchichine[Title/Abstract])) OR (aqua colchin[Title/Abstract])) OR (colchicum[Title/Abstract])) OR (colchily[Title/Abstract])) OR (colchimedio[Title/Abstract])) OR (colchiquim[Title/Abstract])) OR (colchisol[Title/Abstract])) OR (colchysat[Title/Abstract])) OR (colcine[Title/Abstract])) OR (colcrys[Title/Abstract])) OR (colgout[Title/Abstract])) OR (goutichine[Title/Abstract])) OR (goutnil[Title/Abstract])) OR (kolkicin[Title/Abstract])) OR (nsc 757[Title/Abstract])) OR (tolchicine[Title/Abstract])  #2: ((((((("osteoarthritis"[MeSH Terms]) OR (osteoarthriti*[Title/Abstract])) OR (osteoarthro*[Title/Abstract])) OR (gonarthriti*[Title/Abstract])) OR (coxarthriti*[Title/Abstract])) OR (coxarthro*[Title/Abstract])) OR (osteo?arthritis[Title/Abstract])) OR (gonarthro*[Title/Abstract])  #3:((compar*[tiab]) OR ((singl*[tiab] or doubl*[tiab] or tripl*[tiab]) and (mask*[tiab] or blind*[tiab]))) OR (random*[tiab] or placebo[tiab] or controlled[tiab] or trial*[tiab])  #4: #1 AND # 2 AND #3 |
| --- | --- |
| Embase | #1: 'colchicine'/exp OR 'colchicine' OR colcemid*:ab,ti OR demecolcine:ab,ti OR colchamine:ab,ti OR lumicolchicine*:ab,ti OR (((beta OR gamma) NEXT/1 lumicolchicine*):ab,ti) OR colchicin*:ab,ti OR colchichine:ab,ti OR 'aqua colchin':ab,ti OR colchicum:ab,ti OR colchily:ab,ti OR colchimedio:ab,ti OR colchiquim:ab,ti OR colchisol:ab,ti OR colchysat:ab,ti OR colcine:ab,ti OR colcrys:ab,ti OR colgout:ab,ti OR goutichine:ab,ti OR goutnil:ab,ti OR kolkicin:ab,ti OR 'nsc 757':ab,ti OR tolchicine:ab,ti  #2: 'osteoarthritis'/exp OR osteoarthriti*:ab,ti OR osteoarthro*:ab,ti OR gonarthriti*:ab,ti OR coxarthriti*:ab,ti OR coxarthro*:ab,ti OR gonarthro*:ab,ti OR osteo*arthritis:ab,ti  #3: ((compar*:ti,ab) OR ((singl*:ti,ab OR doubl*:ti,ab OR tripl*:ti,ab) AND (mask*:ti,ab OR blind*:ti,ab))) OR (random*:ti,ab OR controlled:ti,ab OR trial*:ti,ab OR placebo:ti,ab)  #4: #1 AND # 2 AND #3 |
| Cochrane | #1: MeSH descriptor: [Colchicine] explode all trees  #2: (colcemid*):ti,ab,kw OR (demecolcine):ti,ab,kw OR (colchamine):ti,ab,kw OR (lumicolchicine*):ti,ab,kw OR (gamma‐lumicolchicine*):ti,ab,kw OR (beta‐lumicolchicine):ti,ab,kw OR (colchicin*):ti,ab,kw OR (colchichine):ti,ab,kw OR (aqua next colchin):ti,ab,kw OR (colchicum):ti,ab,kw OR (colchily):ti,ab,kw OR (colchimedio):ti,ab,kw OR (colchiquim):ti,ab,kw OR (colchisol):ti,ab,kw OR (colchysat):ti,ab,kw OR (colcine):ti,ab,kw OR (colcrys):ti,ab,kw OR (colgout):ti,ab,kw OR (goutichine):ti,ab,kw OR (goutnil):ti,ab,kw OR (kolkicin):ti,ab,kw OR (nsc next 757):ti,ab,kw OR (tolchicine):ti,ab,kw  #3: MeSH descriptor: [Osteoarthritis] explode all trees  #4: (osteoarthriti*):ti,ab,kw OR (osteoarthro*):ti,ab,kw OR (gonarthriti*):ti,ab,kw OR (coxarthriti*):ti,ab,kw OR (coxarthro*):ti,ab,kw OR (osteo?arthritis):ti,ab,kw OR (gonarthro*):ti,ab,kw OR (gonarthriti*):ti,ab,kw  #5: (#1 OR #2) AND (#3 OR #4) |
| Web of Science | #1: TS=(osteoarthriti* OR osteoarthro* OR gonarthriti* OR gonarthro*OR coxarthriti* OR coxarthro* OR osteo*arthritis)  #2: TS=(colchicine OR colcemid* OR demecolcine OR colchamine OR lumicolchicine* OR gamma‐lumicolchicine* OR beta‐lumicolchicine OR colchicin* OR aqua colchin OR colchicum OR colchily OR colchimedio OR colchiquim OR colchisol OR colchysat OR colcine OR colcrys OR colgout OR goutichine OR goutnil OR kolkicin OR nsc 757 OR tolchicine)  #3: TS=(((compar*) OR ((singl* OR doubl* OR tripl*) AND (mask* OR blind*))) OR (random* OR controlled OR trial* OR placebo))  #4: #1 AND # 2 AND #3 |
